# Observational study of coagulation activation in early breast cancer: development of a prognostic model based on data from the real world setting

**DOI:** 10.1186/s12967-018-1511-x

**Published:** 2018-05-16

**Authors:** Chiara Mandoj, Laura Pizzuti, Domenico Sergi, Isabella Sperduti, Marco Mazzotta, Luigi Di Lauro, Antonella Amodio, Silvia Carpano, Anna Di Benedetto, Claudio Botti, Francesca Ferranti, Anna Antenucci, Maria Gabriella D’Alessandro, Paolo Marchetti, Silverio Tomao, Giuseppe Sanguineti, Antonio Giordano, Marcello Maugeri-Saccà, Gennaro Ciliberto, Laura Conti, Patrizia Vici, Maddalena Barba

**Affiliations:** 1 Department of Clinical Pathology, IRCCS Regina Elena National Cancer Institute, Via Elio Chianesi 53, 00144 Rome, Italy; 2 Division of Medical Oncology 2, IRCCS Regina Elena National Cancer Institute, Via Elio Chianesi 53, 00144 Rome, Italy; 3 Biostatistics Unit, IRCCS Regina Elena National Cancer Institute, Via Elio Chianesi 53, 00144 Rome, Italy; 4 Medical Oncology Unit Policlinico Sant’Andrea, Via di Grottarossa 1035/1039, 00189 Rome, Italy; 5 Department of Pathology, IRCCS Regina Elena National Cancer Institute, Via Elio Chianesi 53, 00144 Rome, Italy; 6 Department of Surgery, IRCCS Regina Elena National Cancer Institute, Via Elio Chianesi 53, 00144 Rome, Italy; 7 Radiology Department, IRCCS Regina Elena National Cancer Institute, Via Elio Chianesi 53, 00144 Rome, Italy; 8 Department of Medico-Surgical Sciences and Biotechnologies, University of Rome “Sapienza”, Corso della Repubblica 79, 04100 Latina, Italy; 9 Department of Radiotherapy, IRCCS Regina Elena National Cancer Institute, Via Elio Chianesi 53, 00144 Rome, Italy; 10 Sbarro Institute for Cancer Research and Molecular Medicine e del Center for Biotechnology, College of Science and Technology, Temple University, 1900 N, 12th Street, Philadelphia, PA USA; 11 Scientific Direction, IRCCS Regina Elena National Cancer Institute, Via Elio Chianesi 53, 00144 Rome, Italy

**Keywords:** Early breast cancer, Coagulation activation, Prognostic score, Survival

## Abstract

**Background:**

Cancer and coagulation activation are tightly related. The extent to which factors related to both these pathologic conditions concur to patient prognosis intensely animates the inherent research areas. The study herein presented aimed to the development of a tool for the assessment and stratification of risk of death and disease recurrence in early breast cancer.

**Methods:**

Between 2008 and 2010, two hundreds thirty-five (N: 235) patients diagnosed with stage I–IIA breast cancer were included. Data on patient demographics and clinic-pathologic features were collected in course of face-to-face interviews or actively retrieved from clinical charts. Plasma levels of plasminogen activator inhibitor type 1 (PAI-1), fragment 1 + 2 (F1 + 2), thrombin antithrombin complex (TAT), factor VIII (FVIII), and D-dimer (DD) were measured at breast cancer diagnosis and prior to any therapeutic procedure, including breast surgery. The risk of death was computed in terms of overall survival (OS), which was the primary outcome. For a subset of patients (N = 62), disease free survival (DFS) was also assessed as a measure of risk of disease recurrence.

**Results:**

Median follow up was 95 months (range 6–112 months). Mean age at diagnosis was 60.3 ± 13.4 years. Cancer cases were more commonly intraductal carcinomas (N: 204; 86.8%), pT1 (131; 55.7%), pN0 (141; 60%) and G2 (126; 53.6%). Elevated levels of PAI-1 (113; 48.1%) represented the most frequent coagulation abnormality, followed by higher levels of F1 + 2 (97; 41.3%), DD (63; 27.0%), TAT (34; 40%), and FVIII (29; 12.3%). In univariate models of OS, age, pT, DD, FVIII were prognostically relevant. In multivariate models of OS, age (p = 0.043), pT (p = 0.001), levels of DD (p = 0.029) and FVIII (p = 0.087) were confirmed. In the smaller subgroup of 62 patients, lymph node involvement, percent expression of estrogen receptors and levels of FVIII impacted DFS significantly.

**Conclusions:**

We developed a risk assessment tool for OS including patient- and cancer-related features along with biomarkers of coagulation activation in a cohort of early BC patients. Further studies are warranted to validate our prognostic model in the early setting and eventually extend its application to risk evaluation in the advanced setting for breast and other cancers.

**Electronic supplementary material:**

The online version of this article (10.1186/s12967-018-1511-x) contains supplementary material, which is available to authorized users.

## Background

In recent years, the body of knowledge supporting the mutual association between coagulation disorders and cancer has notably grown [1, 2]. Cancer is a widely accepted predisposing factor for thromboembolic events. At the general population level, these events show an incidence rate of one to two per 1000 people/year, while patients with malignancies generally exhibit a 4–10 times greater risk, which may further increase particularly in pancreatic and brain cancer patients [3–5]. Beyond the primitive cancer site, several clinicopathologic features and administered treatments have been consistently described as specific determinants of venous thromboembolism (VTE) in patients with cancers [6, 7].

On the other side of the medal, biomarkers related to coagulation disorders have shown prognostic relevance in lung, colorectal, ovarian cancer and glioblastoma, independently on the occurrence of VTE [8–11]. When focusing on breast malignancies, rapidly growing evidence comes from both the advanced and early setting. In 84 patients with metastatic disease, pre-treatment plasma levels of D Dimer (DD) were positively associated with prognostically relevant clinicopathologic features and circulating levels of cytokines related to angiogenesis [12]. More recently, circulating tumour cells (CTC) have been linked to plasma DD levels in patients with metastatic breast cancer. This latter study also confirmed the previously described association between CTC and VTE [12, 13]. Evidence from the early setting is also intriguing. In a case–control study of genotypic and phenotypic variables related to the tissue factor (TF) pathway, DD levels beyond the 90th percentile were associated with cancer status, with results being not specific to the different subsets of patients as defined by hormone receptor (HR) and HER2 status [14]. In 360 HR negative early breast cancer patients, positive staining at the immunohistochemical assessment of D2-40 and factor VIII (FVIII) was associated with less favorable survival outcomes both in the overall cohort and in patient subgroups [15]. In addition, in a case series including 100 women having undergone breast surgery due to newly diagnosed invasive breast cancer, circulating levels of FVIII were significantly associated with axillary lymph node involvement, number of metastatic nodes, and HER2 status [16].

Based on the previously cited work and institutional experience of dedicated scientists operating in the management of thrombosis in cancer, we have gained increasing awareness of the need of considering biomarkers related to coagulation disorders in the evaluation of treatment outcomes in breast cancer. We have thus focused on the development of an operating tool for risk assessment based on the combined evaluation of patient- and cancer-related features along with biomarkers of coagulation disorders. Among these latter biomarkers, we have specifically focused on plasmatic factors which play a relevant role in terms of activators of blood coagulation. To this aim, we have conceived and conducted a monocentric observational study in a cohort of early breast cancer patients diagnosed and treated at our institution between 2008 and 2010.

## Methods

### Patients and setting

We analyzed records related to two hundreds thirty-five (N: 235) patients diagnosed with stage I–IIA breast cancer and treated at the IRCCS Regina Elena National Cancer Institute of Rome (IRE) between 2008 and 2010. For all of them, data on demographics and key clinicopathologic features were actively retrieved by ad hoc trained personnel. In addition, plasma levels of plasminogen activator inhibitor type 1 (PAI-1), prothrombin fragments 1 + 2 (F1 + 2), thrombin antithrombin complex (TAT), FVIII, and DD were measured at baseline prior to any therapeutic procedure, including surgery. For a subset of these patients (N: 62), data on anticancer systemic treatment were made available and analyzed in reference to the outcomes of interest. Disease free survival (DFS) and overall survival (OS) were computed as the time elapsed between the histologically codified diagnosis performed in surgical specimen and disease progression or death from any cause, and the time from diagnosis to death due to any cause, respectively. The study was conducted in accordance with the Declaration of Helsinki and approved by the IRE Institutional Review Board (IRB). For each participating woman, a written informed consent was secured in case of patient acceptance following invitation to adhere. This study is compliant with the REMARK guidelines, in that it provides relevant information concerning its design, underlying hypothesis, characteristics of the included patients and collected specimen, assay methods, and statistical analysis [17].

### Blood sampling and biomarker assessment

Blood samples were drawn from the antecubital veins and collected in 3.2% sodium citrate vacutainer^®^ vacuum tubes (Vacutainer, Becton–Dickinson, Franklin Lakes, NJ USA). Identical blood collecting procedures were applied to the samples from a control group including 150 cancer-free women seeking blood screening tests at the IRE. The two groups were comparable by age and date at assessment (data available upon request). Blood samples were centrifuged at 2500*g* for 20 min to obtain platelet-poor plasma. Plasma levels of DD and FVIII were immediately assayed by clotting, chromogenic and immunological methods on fully-automated ACLTOP analyzer using HemosIL^®^ commercial kits (Instrumentation Laboratory Company, Bedford, MA USA). Plasma samples were then separated and stored into multiple aliquots at − 80 °C for subsequent testing. Plasma levels of TAT and F1 + 2) were measured by enzyme-linked immunosorbent assay Enzygnost^®^ TAT micro and Enzygnost^®^ F1 + 2 mono kits, respectively (Siemens Healthcare Diagnostics Inc, NY USA), according to the manufacturer instructions. Both assays employ the quantitative sandwich enzyme immunoassay technique. All samples showing values above the standard curve were re-tested with appropriate dilutions. Plasma levels ofPAI-1 were determined by Asserachrom^®^ kit (DiagnosticaStago, Asnieres, France), according to the manufacturer instructions, employing the quantitative sandwich enzyme immunoassay technique.

### Statistical analysis

Descriptive statistics were computed for all the variables of interest. Means and standard deviations (SD) were used to describe age in years, circulating levels of coagulation activators and define cut off points discriminating between case and control patients. Categorical variables were addressed by χ^2^ test or Fisher’s exact test, according to the size and number of groups compared, i.e., two or more than two, respectively. Disease-free Survival (DFS) and OS were calculated by the Kaplan–Meier product limit method. The log-rank test was used to assess differences between subgroups. Significance was set at a p value less than 0.05. The hazard ratio (HR) and the 95% confidence intervals (95% CI) were estimated for each variable using the Cox univariate model. The following variables were tested in univariate analyses: age, tumor size at the post surgical assessment (pT), pathologic loco-regional nodal involvement (pL), grading (G), estrogen/progestin receptor (ER/PgR) expression, HER2 status, molecular subtype (triple negative, luminal A, luminal B, HER2 enriched breast cancer subgroups), percentage of ki-67 expression (ki-67%). The coagulation biomarkers tested were as it follows: PAI-1, f 1 + 2, TAT, FVIII and DD. Variables testing significant in univariate analysis were included in multivariate models using the stepwise regression (forward selection, enter limit and remove limit, p = 0.10 and p = 0.15, respectively). The outcome predictors identified on the basis of the multivariate analysis were then used for prognostic score assessment. The log-HR obtained from the Cox model was used to derive weighting factors of a continuous prognostic index, aimed to identify differential risks for the outcome of interest [18]. Risk classes were derived using the maximally selected log-rank statistics analysis for the best ‘splitter’ cut-off definition [19]. The SPSS (version 21.0; SPSS, Inc., Chicago, IL) and R-Software (version 3.4.2) were used for statistical analyses.

## Results

The median follow up for the overall cohort was 95 months (6–112). The descriptive characteristics of our study participants are displayed in Table 1. Mean age at diagnosis and related SD were 60.3 ± 13.4. As expected, the most commonly represented histology was intraductal carcinoma (204; 86.8%). Cancer cases were most commonly pT1 (131; 55.7%), pN0 (141; 60%) and G2 (126; 53.6%). Overall, based on ER/PgR expression, HER2 status, and ki-67%, the number and percentages of luminal breast cancers, triple negative (TN) and HER2 enriched cases were 187 (79.5%), 30 (12.8%) and 18 (7.7%), respectively.

**Table 1:** Clinical-pathological characteristics of the study participants (N: 235)

Characteristics	Number (N) and percentage (%)
Age (years)	N (%)
Mean (SD)	60.3 (13.4)
Histology	
Intraductal carcinoma	204 (86.8)
Lobular carcinoma	14 (6.0)
Other	9 (3.8)
Unknown	8 (3.4)
pT stage	
pT1	131 (55.7)
pT2	104 (44.3)
pN stage	
pN0	141 (60.0)
pN1	83 (35.3)
Unknown	11 (4.7)
Grading	
1	16 (6.8)
2	126 (53.6)
3	64 (27.2)
Unknown	29 (12.3)
Estrogen receptor status	
Positive	186 (79.1)
Negative	49 (20.9)
Progesterone receptor status	
Negative	71 (30.2)
Positive	164 (69.8)
HER2 status	
Positive	39 (16.6)
Negative	196 (83.4)
% Ki-67	
≤ 15	142 (60.4)
> 15	89 (37.9)
Unknown	4 (1.7)
Molecular subtype	
Triple-negative	30 (12.8)
Luminal A	118 (50.2)
Luminal B	69 (29.3)
HER2-enriched	18 (7.7)

The number and percentage of early breast cancer patients with abnormal levels of biomarkers related to coagulation activation are reported in Table 2. Among our early breast cancer patients, elevated levels of PAI-1 (113; 48.1%) were those most commonly observed, followed by higher levels of F1 + 2 (97;41.3%), DD (63;27.0%), TAT (34;40%), and FVIII (29;12.3%).

**Table 2:** Abnormal levels of biomarkers related to coagulation disorders in our study cohort (N: 235)

Variable	N	Mean (SD)	Patients with abnormal levels of biomarkers N (%)^a^
DD	232	215.13 (174.47)	63 (27.0)
TAT	235	4.94 (3.23)	34 (40.0)
F 1 + 2	235	207.76 (81.18)	97 (41.3)
PAI-1	235	32.46 (23.61)	113 (48.1)
FVIII	235	131.28 (33.65)	29 (12.3)

*PAI-1* plasminogen activator inhibitor type 1, *F1 + 2* fragment 1 + 2, *TAT* thrombin antithrombin complex, *FVIII* factor VIII, *DD* d-dimer

^a^Cut-off values for case discrimination were defined upon the mean + 2SD of each biomarker as assessed in the control group (N: 150)

**Fig. 1:**
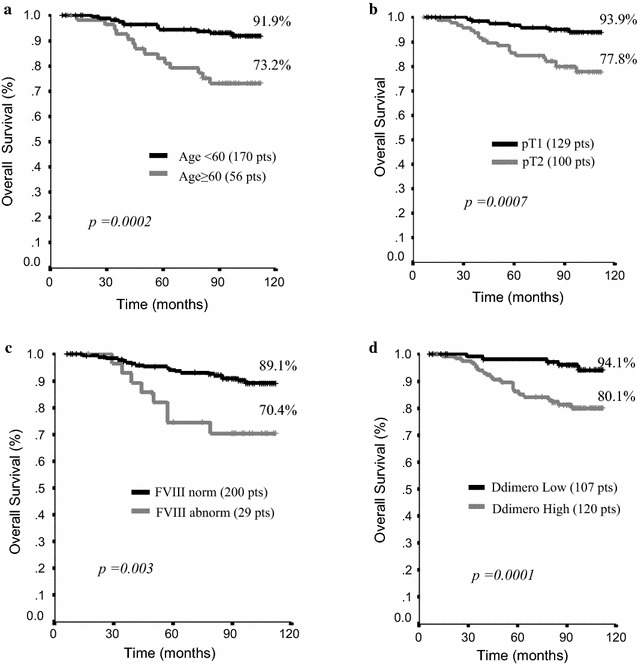
Overall survival by relevant clinical pathologic features and biomarkers of coagulation activation. **a** Overall survival (OS) by age at breast cancer diagnosis. The cut off was defined upon the median age at the study population level; **b** overall survival (OS) by T size as assessed by the pathologist on surgical specimen (pT); **c** overall survival by plasmatic levels of Factor VIII (FVIII). **d** Overall survival (OS) by plasmatic levels of D-Dimer (DD)

Among the factors included in univariate models of OS, those testing significant were age, pT, DD, and FVIII. In brief, breast cancer patients had the highest chances of longer survival if aged 60 years or less (p = 0.0002), showing a pT1 (p = 0.0007), normal levels of FVIII (p = 0.003), and lower levels of DD (p = 0.001) (Fig. 1a–d). In addition, patients with no lymph-node involvement and lower levels of PAI-1 showed longer survival, although not to an extent that was statistically significant (p = 0.05 and p = 0.08, respectively) (data available upon request). In multivariate models, age (p = 0.043), pT (p = 0.001), levels of DD (p = 0.029) and FVIII (p = 0.087) were confirmed as factors of relevant impact on OS. The related HR, 95% CI and p are shown in Table 3.

**Table 3:** Multivariate analysis of factors impacting overall survival (N: 235)

Variable	HR (CI 95%)	p
Age year (> 70 vs ≤ 70)	2.35 (1.026–5.396)	0.043
pT stage (pT2 vs pT1)	4.96 (1.99–12.38)	0.001
DD (high vs low)	3.17 (1.13–8.94)	0.029
FVIII (abnormal vs normal)	2.15 (0.90–5.15)	0.087

*FVIII* factor VIII, *DD* d-dimer

**Fig. 2:**
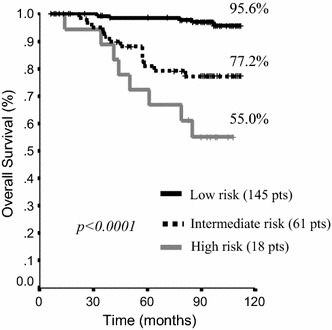
Overall survival (OS) according to risk categories as identified by the nomogram developed based on data from this historic cohort

As mentioned in the “Methods” section, variables testing significant in multivariate analysis were used for prognostic score assessment. Determinants of OS contributed to risk definition and assignment to risk categories according to the best ‘splitter’ cut-off definition as applied to this specific cohort (Table 4). The inherent results are graphically displayed in Fig. 2. In brief, breast cancer patients within the lowest risk category, i.e., with age younger than 70, pT1, circulating levels of FVIII within the normal range, and low levels of DD, showed significantly longer OS compared to the groups of patients at intermediate and high risk, with the related HRs being 95.6, 77.2, and 55.0, respectively (p < 0.0001).

**Table 4:** Prognostic score assessment according to determinants of overall survival in our study cohort (N: 235)

Overall survival	Score points		
	0	1	2
pT	T1	–	T2
FVIII	Normal	Abnormal	–
Age	≤ 70	> 70	–
DD	Low	–	High

*FVIII* factor VIII, *DD* d-dimer

In a subset of patients (N: 62) for whom data related to administered treatments and related outcomes were available, we estimated DFS and OS. Prior to performing such analyses and in order to minimize the chances of selection bias, we compared the sub-cohort of interest (N: 62) and its complement to the overall cohort (N: 173) by relevant characteristics. Results showed statistically significant differences in terms of age when using the cut off value of 70 (p = 0.04), which had been previously defined as the most efficient splitter for the overall cohort. However, when comparing the two subgroups by median age, this difference was no longer observed (55 years within a 35–87 range and 60 within a 29–89 range for the smaller and larger subset, respectively). In the smaller subgroup, we tested the variables included in the scoring tool in multivariate Cox models of DFS, which revealed significant prognostic relevance for lymph node involvement, percent expression of ER and levels of FVIII (Additional file 1: Table S1). However, the low number of recurrences, i.e., 12, prevented us from applying the scoring tool for risk stratification in this subset. Similarly, when considering OS, the number of events was extremely low, i.e., 8 (median OS not reached). This prevented us from performing additional analyses.

## Discussion

We conducted an observational study of 235 early breast cancer patients who were diagnosed and treated at the IRCCS Regina Elena Cancer Institute in the time frame between July 2008 and September 2010. For all of them, blood samples were collected prior to any therapeutic procedures and assessed for circulating levels of biomarkers related to coagulation activation. One hundred fifty cancer free women comparable by age and recruitment period served as control group. Data analysis were planned and performed to allow the development of a tool instrumental to the assessment of risk categories based on features related to relevant clinicopathologic characteristics and coagulation profiling for the biomarkers of interest. Within our study population, results from multivariate analysis revealed a prognostic role for age, pT, circulating levels of FVIII and DD. These variables were then used to define risk categories, with cut off points obtained by applying the best “splitter” cut-off definition to our case-series. The so developed tool proved efficacy in distinguishing categories characterized by significantly different survival estimates. In more detail, the lowest risk of death from any cause was ascribed to patients aged 70 years or less, with a pT1 disease at diagnosis, circulating levels of FVIII within the normal range and low levels of DD. Age older than 70 years and FVIII levels greater than the pre-established cut off values where instead associated with an intermediate risk of death, while the highest risk of death was associated with a pT2 and DD levels beyond the threshold defined for our study population.

Among the clinically relevant patient- and disease-related determinants of interest, age and tumour size (pT) showed prognostic relevance in uni- and multi-variate analysis and were thus included in the model for risk stratification. This evidence is consistent with previous literature from the early setting [20, 21]. In strict regard to the patient age, our results provide several hints for discussion. In our case series, values of median age and best “splitter” cut-off for age were 60 and 70 years, respectively. In addition, the outcome considered for the overall case series was death from any cause as we lacked data on breast cancer specific mortality for the totality of our patients, as specifically pointed out in the paragraph concerning this study limitations. Thus, we addressed an outcome, i.e., death from any cause, which is definitely affected by aging. Indeed, the lack of specific data concerning the extent to which age, co-morbidities, and breast cancer have concurred to determine our patients’ death may be more appropriately exemplified by the use of a terminology distinguishing between “likely cause of death” vs “other causes of death”, particularly in light of the broad age range which characterizes our case-series, i.e., 29–39 years. The relation between aging and breast cancer is complex and the investigation of the underlying mechanisms animates intensely the inherent research area [22]. In reference to recently published and clinically focused evidence, Lodi et al. have evaluated relevant clinicopathologic features and breast cancer specific survival outcomes in a systematic review of women over 70 years with breast cancer. Sixty-three original studies published between 2006 and 2016 were considered. Consistently with our findings, the authors reported on the association between older age and significantly higher 5- and 10-year mortality [23]. Older age at breast cancer diagnosis should be considered not only in light of its prognostic role for the disease of interest, but also in reference to the role of DD and FVIII as biomarkers of aging, widely and consistently supported by the inherent literature in both non-cancer and cancer patients [24–28]. On this basis and in strict regard to our study population, we assessed the interaction between age and circulating levels of DD and FVIII in Cox models including an interaction term. In this specific cohort, we could not observed significant interaction between the variables tested (p = 0.20 and p = 0.94, for the interaction between age and DD and FVIII, respectively).

In this study population, we found no evidence supporting the prognostic relevance of the specific molecular subtype, i.e., luminal A, luminal B, HER2 enriched and triple negative breast cancer, on patient survival. Indeed, in multivariate analysis of OS, the related variables tested not significant (p: 0.74). This finding, i.e., lack of the impact of molecular subtype on the outcomes of interest in a breast cancer patient population from the early setting, is consistent with previous studies [14, 16] and in need of further assessment for clarification purposes. Indeed, in our case series, we exclusively observed some evidence of the prognostic relevance of ER expression, one of the main determinants of the specific molecular subgroups, in the subset of patients for whom DFS data were available (N: 62), with our results supporting a protective role of ER expression (p: 0.003). This same patient subgroup also offered the chance for evaluating our scoring tool in reference to DFS. Although somewhat limited by the restricted sample size, results from the analysis performed within this subset confirm the prognostic relevance of biomarkers related to coagulation disorders, and the need for including such evidence in risk assessment for early breast cancer patients.

An appropriate discussion of our results cannot exclude a referral to the existing evidence concerning the use of anticoagulants in cancer patients, which has been recently summarized in a Cochrane systematic review carried out by Kahale et al. In brief, the authors conducted a comprehensive literature search updated to December 2017. Of the identified 7668 unique citations, 16 manuscripts reporting on 7 randomized clinical trials (RCTs) fulfilled the inclusion criteria and were thus included. Overall, these trials enrolled 1486 participants. Results from the meta-analyses of the RCTs included do not rule out a mortality benefit from oral anticoagulation in people with cancer but suggest an increased risk for bleeding. In the attempt to interpret these findings correctly, the lack of data specifically referred to the site of cancer origin should be considered. Indeed, the need of further evidence specifically related to the cancer type and stage is acknowledged by the authors themselves when discussing their research implications [29].

The pathogenetic layout of the association between the activation of coagulation and cancer is multifactorial in nature. In addition, most of the actors involved play a pivotal role in several mechanistic pathways that sustain cancer-related biological processes with a notable degree of overlap. The previously mentioned role of FVIII and DD as factors involved both in cancer, thrombogenesis and aging may appropriately exemplify this latter assertion [24–28]. Cancer may provide an unusual and polyvalent frame within which patient- and disease-related features concur to outcome determinism, both for thromboembolic and cancer related events. The relationship between thrombosis and cancer is founded on the evidence that cancer promotes a prothrombotic switch of the host hemostatic system, and in turn, blood clotting activation is intimately tight to tumor growth and dissemination. The main mechanisms of cancer-related thrombosis encompass the expression of procoagulant factors at the tumour cell level, the release of microparticles, inflammatory cytokines e.g., tumor necrosis factor-alpha, interleukin-6, and proangiogenic factors, e.g., vascular endothelial growth factor, basic fibroblast growth factor by tumor and/or host cells, and the expression of adhesion molecules to bind platelets, endothelial cells, and leucocytes. These same properties are also involved in cancer progression [30, 31].

Our study has some limitations, which are mainly represented by the lack of data concerning cancer-specific survival for the overall case series. This is unfortunately common when working in the real word setting. Indeed, data collection and entering into dedicated databases has not stably entered the clinical practice. To mitigate such limitation, we have attempted to perform subgroup analysis in a subset of patients for whom cancer-specific survival data were available. Unfortunately, this subset was extremely limited in size (N: 62). This refrained us from conducting analysis beyond the multivariate models. However, also in this smaller subset, we could observe evidence supporting the prognostic relevance of both patient- and cancer-specific feature along with circulating levels of coagulation biomarkers. The lack of data on menopausal status should also be acknowledged, given the relevant differences in terms of risk factors, presentation at diagnosis, characteristics and management between pre- and post-menopausal breast cancer patients [32–34]. In the attempt to minimize such limitation, we codified a categorical variable with a 50-year cut off value and assumed that women aged less than 50 years (N: 70; 25.5%) were most likely premenopausal. However, in univariate models of OS and DFS, our surrogate variable of menopausal status did not test significant (p: 0.32 and p: 0.81, respectively).

Our study also has strengths of relevance. Among them, central management of biomarkers of coagulation activation is noteworthy. Plasma samples were collected and handled according to pre-specified and highly standardized operative procedures. Sample assessment was performed at the institutional laboratories, which are ISO-certified (ISO 9001 certification*).* This increases our confidence in the quality of the evidence stemming from our study. As cited in the “Results” section, the median follow up for the cohort of interest was 95 months, which is fairly acceptable in terms of length when assessing outcomes in a cohort of early breast cancer patients. However, this 10-year follow up window imposes considerations related to the remarkable advances achieved both in the loco-regional and systemic treatment [35–40] and invites caution in the generalization of our results to early breast cancer patients in current treatment. At the same time, this latter matter, along with the results from the work herein presented, encourages future investigation within this same research pipeline.

## Conclusions

We provide evidence in support of the prognostic relevance of age at cancer diagnosis, pT, levels and FVIII and DD in a case-series including 235 stage I-IIA breast cancer patients. The score including these factors proved efficacy in distinguishing risk categories in reference to survival outcomes. Risk assessment and stratification for cancer related outcome deserves active investigation. To this purpose, the subsequent steps to be taken possibly include the validation of our model in independent cohorts of early breast cancer patients participating in adequately sized, ad-hoc, prospective studies. These latter studies should ideally allow the serial assessment of biomarkers of coagulation activation at pre-specified time points. This would allow to monitor these biomarkers in parallel with the disease course and integrate the inherent data with those pertinent to breast cancer treatment and related outcomes. In addition, future studies should also include VTE-related outcomes, e.g., deep vein thrombosis, pulmonary embolism. These strategies may help compute multiple risk estimates for time dependent outcomes, e.g. DFS and OS, and, at the same time, help define more accurately the cause of death. We would also acknowledge the potential use of data concerning the activation of coagulation as measured throughout the levels of circulating biomarkers in informing therapeutic decisions concerning the specific therapy to be administered in the adjuvant setting in light of the increased risk of VTE particularly, though not exclusively, associated with hormonal therapy [41]. The identification of the high-risk subgroups for both cancer- and coagulation-related death and establishment of the most appropriate therapeutic strategies possibly including antithrombotic agents is undeniably tighten to multidisciplinary efforts of medical oncologists and clinical pathologists with a solid background in the prevention, diagnosis and management of cancer patients with prothrombotic alterations. A deeper and, as previously stated, multidisciplinary characterization of cancer patients represents the best prelude to outcome amelioration and correct interpretation. If considering that about half of all cancer patients, and as many as 90% of those from the metastatic setting show abnormalities in one or more coagulation-related parameters [42], a still relevant number of queries concerning the management of these patients remain unsatisfactorily addressed and will hopefully fuel cancer research in the upcoming years.

## Additional file

**Additional file 1: Table S1.** Multivariate analysis for disease-free survival in a subset of 62 breast cancer patients.

## Abbreviations

PAI-1: plasminogen activator inhibitor type 1
F1 + 2: fragment 1 + 2
TAT: thrombin antithrombin complex
FVIII: factor VIII
DD: D dimer
OS: overall survival
DFS: disease free survival
VTE: venous thromboembolism
CTC: circulating tumour cells
TF: tissue factor
HR: hormone receptor
SDs: standard deviations
HR: hazard ratios
TN: triple negative
ER: estrogen receptors
RCTs: randomized clinical trials
