# Supplementary material for: Observational study of coagulation activation in early breast cancer: development of a prognostic model based on data from the real world setting
Source: J Transl Med. 2018 May 16;16:129. doi: 10.1186/s12967-018-1511-x (PMC5956941; doi:10.1186/s12967-018-1511-x)
Supplement: Supplementary file 1 — Additional file 1: Table S1. Multivariate analysis for disease-free survival in a subset of 62 breast cancer patients. [file 12967_2018_1511_MOESM1_ESM.docx]

**Additional file 1: Table 1.** Multivariate analysis for Disease-free Survival in a subset of 62 breast cancer patients.

| **Variable** | **HR (CI95%)** | **p** |
| --- | --- | --- |
| pN (pos vs neg) | 4.69 (1.37-15.98) | 0.01 |
| ER (neg vs pos) | 9.44 (2.18-40.85) | 0.003 |
| FVIII (abnormal vs normal) | 12.42 (2.52-61.29) | 0.002 |

ER: Estrogen Receptors; FVIII: factor VIII.
